# Meningioma with rhabdoid, papillary and clear cell features: case report and review of association of rare meningioma variants 

**DOI:** 10.5414/NP300408

**Published:** 2011-10-18

**Authors:** F Rogerio, V. de Araújo Zanardi, J. Ribeiro de Menezes Netto, L. de Souza Queiroz

**Affiliations:** 1 Department of Pathology; 2 Department of Radiology, Faculty of Medical Sciences, University of Campinas – Unicamp, Campinas, SP, Brazil

**Keywords:** meningioma, rhabdoid, papillary, clear cell, immunohistochemistry

## Abstract

Meningiomas are common central nervous system tumors with a wide range of morphological variants, assigned World Health Organization (WHO) Grades I – III. We report an extremely rare rhabdoid, papillary and clear cell meningioma (WHO Grade III) in a 29-year-old female, who presented with diplopia and headache over a few days, 2 years ago. Magnetic resonance imaging showed a well-circumscribed, lobulated, predominantly solid and contrast-enhancing lesion in the right temporal, parietal and occipital lobes. On routine staining, the tumor did not display classical meningioma features. A wide immunohistochemical panel ruled out metastasis and endorsed the meningothelial nature of the lesion (positivity for epithelial membrane antigen and vimentin). Electron microscopy did not show usual hallmarks of meningioma but was helpful in excluding other tumors. Even though the three variants are associated with aggressive behavior, the patient is currently asymptomatic. The concurrent use of different techniques was essential for diagnosis.

## Introduction 

The vast majority of meningiomas are benign tumors assigned Grade I according to the World Health Organization (WHO) and belong to one or more of the following histological patterns: meningothelial, fibrous, transitional, psammomatous, angiomatous, microcystic, secretory, lymphoplasmacyte-rich and metaplastic. Four other types are associated with less favorable prognosis and are considered WHO Grade II (chordoid and clear cell) or III (papillary and rhabdoid). These higher-grade tumors are prone to recur, metastasize and/or are related to shorter survival times [1]. 

Association of different histological patterns in the same lesion is common in low grade meningiomas [1] but far less frequent in high-grade variants such as tumors combining papillary and rhabdoid features [2, 3, 4, 5, 6, 7]. Here we report the unusual coexistence of three high-grade variants (rhabdoid, papillary and clear cell), with emphasis on immunohistochemical and ultrastructural findings, and review the pertinent literature. 

## Case history and methods 

**Figure 1.**
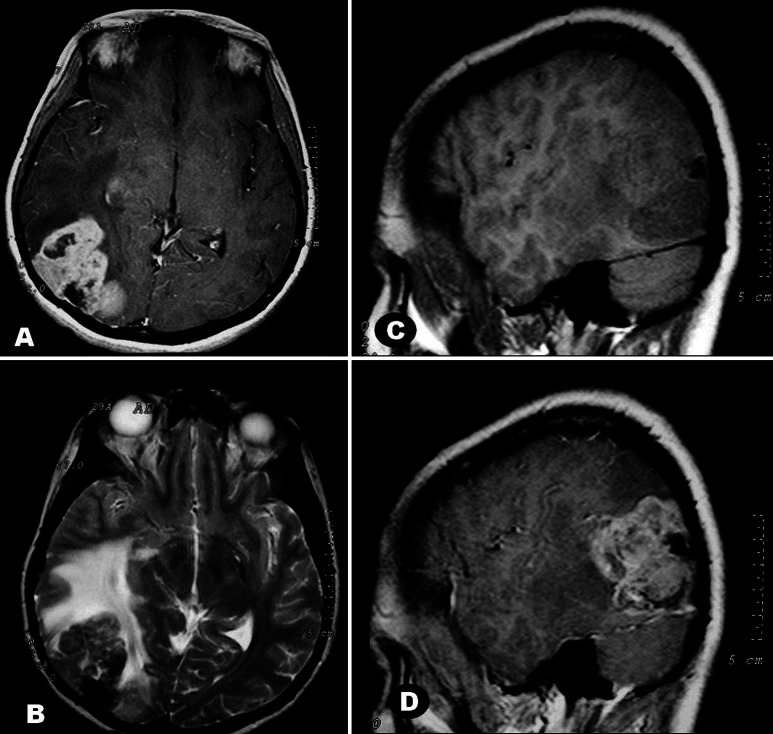
A: Large, lobulated, irregular mass partially occupying the posterior temporal and occipital lobes of the right cerebral hemisphere, with extensive dural attachment. The lesion is solid with cysts and enhances strongly in this T1 weighted image (WI) after gadolinium administration. B: Tumor is isointense to brain in T2WI. The posterior area with signal loss is probably calcified. Marked edema of the hemispheric white matter is noted by its hyperintensity. C, D: Parasagittal sections in a T1 weighted study, before (C) and after (D) contrast. In C, note isointensity of tumor to cerebral cortex and hipointensity of surrounding white matter due to edema.

A 29-year-old female was admitted with diplopia and occipital headache over 4 days. Physical examination revealed deficit of the lateral rectus muscle of the right eye, neck stiffness and bilateral papilledema. Magnetic resonance imaging (MRI) showed a voluminous tumor spanning the posterior right temporal, inferior parietal and occipital lobes. The lesion was irregularly lobulated with uneven borders, isointense on T1- and T2-weighted images, and enhanced strongly, albeit heterogeneously, after contrast. A calcified area was seen in the posterior part of the tumor close to dural attachment. Marked edema of white matter and mass effect were noted (Figure 1). No other intracranial lesions were apparent. The tumor was totally removed through right occipital craniotomy. Retrospectively she reported no family history of central or peripheral nervous system tumors. 

Tumor tissue was fixed in 10% neutral buffered formalin and processed for paraffin embedding. 5 µm thick sections were stained with hematoxylin and eosin (H&E). Immunohistochemical analyses using streptavidin-biotin peroxidase complex method were performed with the following antibodies: cytokeratin 7 (CK7; Dako, cat# M7018; 1 : 100), cytokeratin 20 (CK20; Dako, cat# M7019; 1 : 100), cytokeratin pool (AE1AE3; Dako, cat# M3515; 1 : 200); epithelial membrane antigen (EMA; Dako, cat# M0613 1 : 100), carcinoembryonic antigen (CEA; Dako, cat# M0773; 1 : 4,000), synaptophysin (Dako, cat# A0010; 1 : 100), chromogranin (Dako, cat# A0430; 1 : 2,000), glial fibrillary acidic protein (GFAP; Dako, cat# M0761; 1 : 500), smooth muscle actin (1A4; Dako, cat# M0851; 1 : 200), desmin (Dako, cat# M0760; 1 : 50), calcitonin (Dako, cat# A0576; 1 : 300), thyroglobulin (Dako, cat# M0781; 1 : 200), thyroid transcription factor 1 (TTF1; Dako, cat# M0725; 1 : 500), a-fetoprotein (Dako, cat# A0008; 1 : 200), estrogen receptor (ER; Diagnostic Biosystems, cat# MOB195; 1 : 100), progesterone receptor (PR; Diagnostic Biosystems, cat# MOB103-1; 1 : 400), vimentin (Dako, cat# M0725; 1 : 600) and S100 protein (Dako, cat# Z0311; 1 : 3,000). Cellular proliferation was inferred through immunostaining for Ki67 protein (Dako, cat# M7240; 1 : 500). For ultrastructural studies, fragments were glutaraldehyde-fixed, post fixed in osmium tetroxide, block stained with uranyl acetate and embedded in Araldite. Ultrathin sections were contrasted with lead citrate and photographed in a Zeiss EM-10 electron microscope. 

## Results 

Macroscopically, the surgically resected tissue consisted of three nodules that measured 5.0 cm, 3.5 cm and 1.0 cm in their greatest dimension. The specimens were roughly well circumscribed, white or red, with some irregular yellowish areas reminiscent of necrosis, and their consistency varied from soft to compact. 

**Figure 2.**
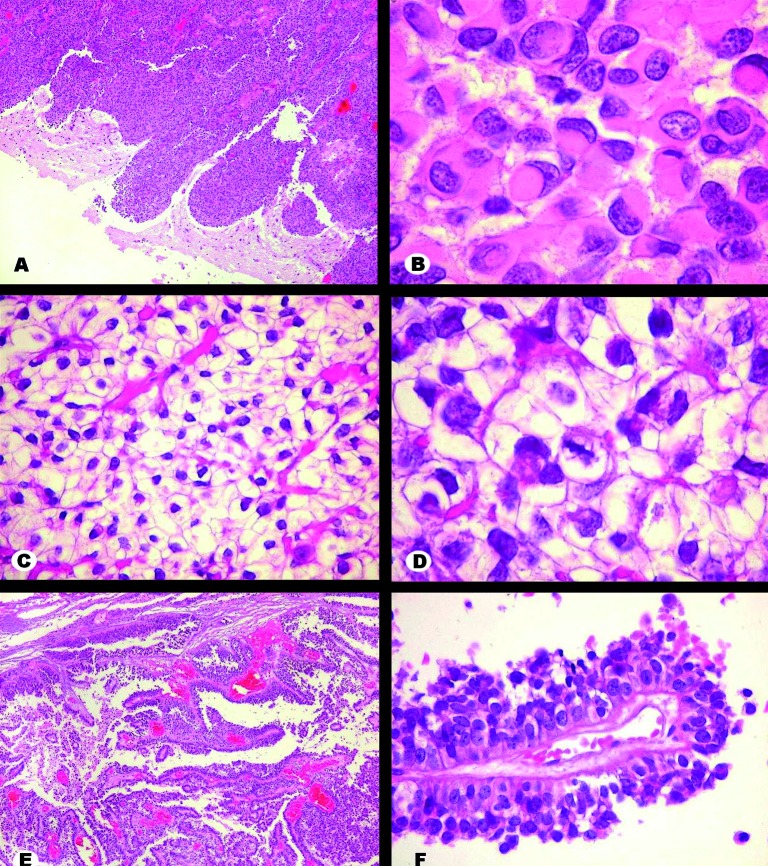
H&E stain. A: Interface between rhabdoid area of tumor and brain. Tumor cells invade surrounding brain as blunt projections. B: Detail of rhabdoid area. Cells show eccentric nuclei indented by a round hyaline corpuscle occupying most of the cytoplasm. C, D: Clear cell area. Cells have abundant pale cytoplasm with distinct borders and regular nuclei. Blood capillaries are thin and well distributed. A mitotic figure is seen in D, at center. E, F: Papillary component. Cells arrange themselves along branched blood vessels creating multilayered papillae. There was tendency for peripheral cells to undergo necrosis, enhancing the pseudopapillary architecture. Original magnification: A × 40, B × 400, C × 100, D × 400, E × 40, F × 100.

Histologically, three patterns were observed in the H&E-stained sections. The first showed rhabdoid morphology and was composed of sheets of cells with eccentric nuclei, often displaying dense chromatin. The cytoplasm was homogenous, hyaline and contained a paranuclear inclusion-like structure. Neoplastic brain invasion was observed as tongue-like bulges or single cells infiltrating the adjacent parenchyma. The second pattern was constituted by polygonal cells showing clear cytoplasm and central nucleus with dense chromatin. Such cells were homogeneous, organized in groups circumscribed by delicate blood vessels and occasionally showed intracytoplasmic glycogen accumulation (PAS-positive, diastase-sensitive material). In the third histological pattern, cells with oval nuclei, dense chromatin and distinct cytoplasmic limits were arranged in perivascular pseudopapillary distribution. Mitotic figures and necrotic foci were common, predominantly in the rhabdoid and clear cell components. In some regions, intermingling of these histological patterns was observed. Whorl formation, a classical feature of meningioma, was hardly ever observed (Figure 2). 

**Figure 3.**
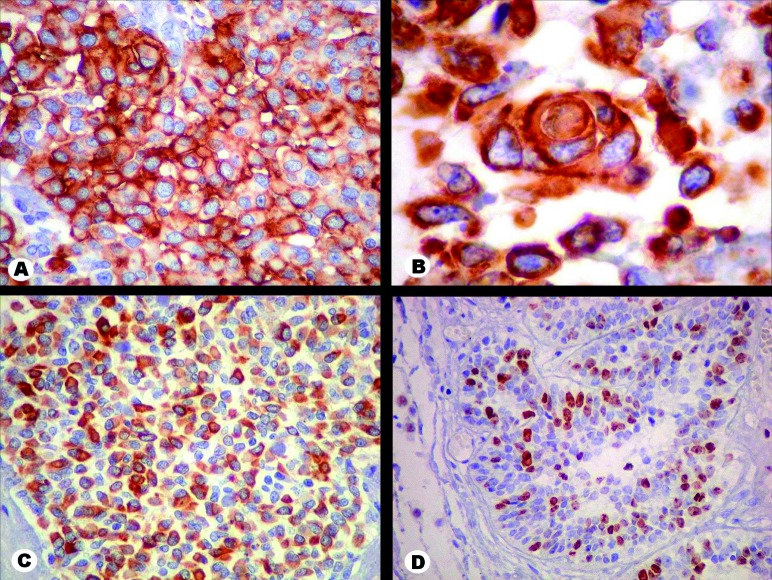
Immunohistochemistry. A: Tumor cells of the rhabdoid area are strongly positive for epithelial membrane antigen (EMA) in the cytoplasm and cell membrane. B: Vimentin marks tumor cell cytoplasm in all areas. An uncommon incipient whorl formation is depicted in a rhabdoid area. C: Labeling of scattered cells by pancytokeratin (AE1AE3), here shown in a rhabdoid region, is known to occur in high grade meningiomas. D: In papillary sections of the tumor, positivity for Ki67 approached 30% of nuclei. Original magnification: A × 100, B × 400, C × 100, D × 100.

Immunostaining was diffuse and intense for vimentin and EMA and focally positive for S100 protein and AE1AE3. About 10% to 30% of the cells were marked for Ki67, according to the area (Figure 3). No positivity was found for CK7, CK20, a-fetoprotein, synaptophysin, chromogranin, calcitonin, thyroglobulin, TTF1, CEA, smooth muscle actin, desmin, GFAP, ER and PR. As the patient had no previous history of neoplasm elsewhere, the findings supported the diagnosis of a meningioma with rhabdoid, papillary and clear cell variants. The tumor was classified as WHO Grade III, due to the rhabdoid and papillary components. 

**Figure 4.**
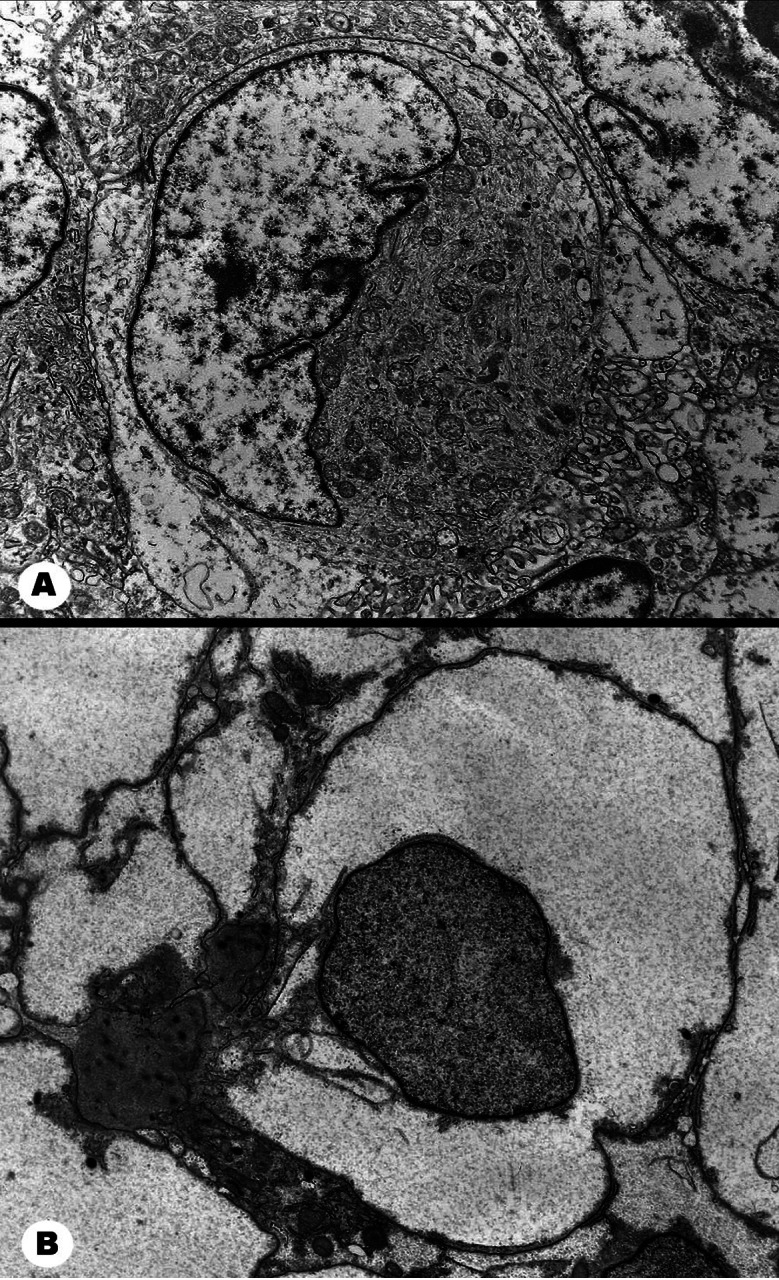
Electron microscopy. A: A rhabdoid cell has its crescent shaped nucleus pushed to the periphery by the abundant cytoplasm with bundles of intermediate filaments mixed with mitochondria. Chromatin is well distributed, the nuclear membrane is thrown into folds and a small nucleolus is apparent. The well defined plasma membrane abuts against neighboring cells. Extracellular fluid and junctional complexes are not visible. B: A clear cell stands out by its abundant, electron lucent, finely granular cytoplasm likely to be glycogen-rich. Very scanty organelles can be discerned. The nucleus is nearly round with stippled chromatin. Cell limits are conspicuous, without junctional complexes and minimal extracellular space. Original magnification: A, B × 5,000.

Ultrastructurally, rhabdoid and clear cell areas were constituted by cells with distinct limits, round or polygonal in shape. Nuclei showed irregular shape and often visible nucleoli, both more conspicuous in the rhabdoid component. Intercellular space, cell membrane interdigitations and junctional complexes were hardly detectable. Rhabdoid cells showed a broad cytoplasm, rich in entangled intermediate filaments that frequently entrapped organelles, presumably corresponding to the inclusion-like structures seen in paraffin sections. Clear cells exhibited cytoplasmic glycogen accumulation, appearing as fine granules amid organelles (Figure 4). 

After pathologic diagnosis of a high-grade meningioma with rhabdoid, papillary and clear cell variants, the patient was submitted to radiation therapy. Two years after surgery, MRI control showed the surgical lacuna in the right posterior temporal and occipitoparietal regions. As the patient developed allergy to gadolinium, further evaluation of tumor progression was hindered. She is presently well and resumed work. 

## Discussion 

We present a rare case of high-grade meningioma with rhabdoid, papillary and clear cell morphologic features in a 29-year-old female. The initial diagnostic difficulty was differentiating between primary lesion and metastatic carcinoma. The latter seemed unlikely in the absence of a known tumor elsewhere and in view of the early age of the patient. Among the primary intracranial lesions, possible differential diagnoses included oligodendroglioma, extraventricular neurocytoma, clear cell ependymoma and a complex meningioma. Oligodendroglioma was considered because of its similarity to the clear cell areas, and for clear cells seen elsewhere, such as in papillary regions. The same might be said of clear cell ependymoma, which, in addition, features perivascular pseudorosettes observed in some areas. However, negativity for GFAP ruled out both. Extraventricular neurocytoma was excluded because of negativity for synaptophysin. Negativity for CK7, CK20, a-fetoprotein and for some primary site-related antigens (thyroid, lung and breast) did not support the hypothesis of metastatic carcinoma. Immunopositivity for EMA and vimentin in association with the imaging findings established the final diagnosis of meningioma. Labeling of many cells for the cytokeratin pool AE1AE3 is not in conflict with that conclusion, as it may be positive in up to 75% of malignant meningiomas [8]. 

Mixed meningiomas composed of rhabdoid, papillary and clear cell variants are extremely unusual. To our knowledge, there is only one paper describing meningiomas with the morphological features shown here. Wu et al. [7] reported three cases of rhabdoid papillary meningiomas with clear cells. All lesions showed histopathologic findings associated with aggressive behavior, such as increased cellularity, small cells with a high nuclear : cytoplasmic ratio, sheet-like growth, necrosis and elevated mitotic and MIB-1 indices. Tumors recurred in 2 of 3 patients at 9 months and 7 years respectively. Such clinical outcome would mainly be determined by the papillary and rhabdoid variants (WHO Grade III). Although our patient had morphologic features similar to the above, we currently have neither clinical nor radiological evidence of recurrence after 2 years. 

“Rhabdoid meningioma” was a term first used by Perry et al. [9] in a series of 15 aggressive meningiomas with rhabdoid features. Some also showed papillae. A similar association between aggressive behavior and meningothelial tumors showing rhabdoid and papillary appearance was described by Kepes et al. [10]. In addition to the rhabdoid and papillary features, our specimen also presented a clear cell component, another variant with unfavorable prognosis assigned Grade II by the WHO. The rarity of such association may be due to the fact that clear cell meningiomas tend to occur only in pure form [9]. 

Regarding the pathologic diagnosis of the unusual meningioma presented here, the employment of different techniques was paramount. H&E-stained sections failed to detect the morphologic hallmarks of a classic meningioma such as whorls and psammoma bodies, the frequency of which has been reported to vary widely in rare meningiomas [5, 7, 9, 10, 11]. Possible hypotheses are undersampling and substitution of classic meningioma by neoplastic tissue with unspecific features, for example, rhabdoid morphology [10]. 

Immunostaining for vimentin and EMA reinforced the meningothelial nature of this atypical lesion, not only in the present case but in other unusual intracerebral tumors with regions reminiscent of meningioma [5, 7, 9, 10]. 

Electron microscopy of our case did not show structures typical of meningiomas, such as desmosomes or interdigitating cytoplasmic processes but helped to characterize both rhabdoid and clear cell components and rule out differential diagnoses, mainly clear cell ependymoma by the absence of cilia or microvilli. Therefore, we only partially agree with Al-Sarraj et al. [12], who claimed the ultrastructural analysis as essential for diagnosing papillary meningioma. 

In conclusion, we report an unusual and potentially aggressive meningioma showing rhabdoid, papillary and clear cells features. Pathological diagnosis was not straightforward in routine stains, but was based on the correlative analysis of clinical, neuroimaging, histopathological, immunohistochemical and ultrastructural findings. The concurrence of different diagnostic methods is fundamental to support the diagnosis, exclude other tumors and define the therapeutic approach. 

## References

[1] Perry A Louis DN Scheithauer BW Budka H Deimling von A Meningiomas. In: Louis DN Ohgaki H Wiestler OD Cavenee WK (eds). WHO Classification of tumours of the central nervous system. Lyon: IARC; 2007 p. 167-172

[2] Hojo H Abe M Rhabdoid papillary meningioma. Am J Surg Pathol. 2001; 25: 964–969 doi:10.1097/00000478-200107000-00018 11420471 10.1097/00000478-200107000-00018

[3] Saito A Nakazato Y Yoshii Y Hyodo A Harakuni T Toita T Ogawa K Horikawa K Terada Y Kinjo S Minei S Anaplastic meningioma with papillary, rhabdoid, and epithelial features: a case report. Brain Tumor Pathol. 2001; 18: 155–159 doi:10.1007/BF02479430 11908873 10.1007/BF02479430

[4] Al-Habib A, Lach B Al Khani A Intracerebral rhabdoid and papillary meningioma with leptomeningeal spread and rapid clinical progression. Clin Neuropathol. 2005; 24: 1–7 15696777

[5] Wakabayashi K Suzuki N Mori F Kamada M Hatanaka M Rhabdoid cystic papillary meningioma with diffuse subarachnoid dissemination. Acta Neuropathol. 2005; 110: 196–198 doi:10.1007/s00401-005-1037-1 15981015 10.1007/s00401-005-1037-1

[6] Santhosh K Kesavadas C Radhakrishnan VV Thomas B Kapilamoorthy TR Gupta AK Rhabdoid and papillary meningioma with leptomeningeal dissemination. J Neuroradiol. 2008; 35: 236–239 doi:10.1016/j.neurad.2008.01.079 18325590 10.1016/j.neurad.2008.01.079

[7] Wu YT Ho JT Lin YJ Lin JW Rhabdoid papillary meningioma: A clinicopathologic case series study. Neuropathology. 2011; doi:10.1111/j.1440-1789.2011.01201.x 10.1111/j.1440-1789.2011.01201.x 21382093

[8] Liu Y Sturgis CD Bunker M Saad RS Tung M Raab SS Silverman JF Expression of cytokeratin by malignant meningiomas: diagnostic pitfall of cytokeratin to separate malignant meningiomas from metastatic carcinoma. Mod Pathol. 2004; 17: 1129–1133 doi:10.1038/modpathol.3800162 15133478 10.1038/modpathol.3800162

[9] Perry A Scheithauer BW Stafford SL Abell-Aleff PC Meyer FB “Rhabdoid” meningioma: an aggressive variant. Am J Surg Pathol. 1998; 22: 1482–1490 doi:10.1097/00000478-199812000-00005 9850174 10.1097/00000478-199812000-00005

[10] Kepes JJ Moral LA Wilkinson SB Abdullah A Llena JF Rhabdoid transformation of tumor cells in meningiomas: a histologic indication of increased proliferative activity: report of four cases. Am J Surg Pathol. 1998; 22: 231–238 doi:10.1097/00000478-199802000-00012 9500225 10.1097/00000478-199802000-00012

[11] Jansen JC Turner J Sheehy J Fagan PA A. Recurrent rhabdoid meningioma: Case report. Skull Base. 2003; 13: 51–54 doi:10.1055/s-2003-37553 15912159 10.1055/s-2003-820557 PMC1131829

[12] Al-Sarraj S King A Martin AJ Jarosz J Lantos PL Ultrastructural examination is essential for diagnosis of papillary meningioma. Histopathology. 2001; 38: 318–324 doi:10.1046/j.1365-2559.2001.01128.x 11318897 10.1046/j.1365-2559.2001.01128.x

